# Activities of Daily Living, Playfulness and Sensory Processing in Children with Autism Spectrum Disorder: A Spanish Study

**DOI:** 10.3390/children8020061

**Published:** 2021-01-20

**Authors:** Nuria Yela-González, Montserrat Santamaría-Vázquez, Juan Hilario Ortiz-Huerta

**Affiliations:** 1 Apace Burgos, 09003 Burgos, Spain; nuriayela96@gmail.com; 2 Health Sciences Department, Universidad de Burgos, 09001 Burgos, Spain; jhortiz@ubu.es

**Keywords:** sensory integration, play, daily life, sensory disorders, child

## Abstract

The purposes of the study were to identify whether differences exist between Spanish children with ASD and neurotypical development in relation to Activities of Daily Living (ADL), playfulness, and sensory processing; as well as to confirm whether a relation exists between those areas and sensory processing. Study Design: A descriptive cross-sectional study. Methods: Forty children, 20 with a diagnosis of ASD and 20 with neurotypical development, were recruited. The measurement tools used were the Pediatric Evaluation of Disability Inventory (PEDI), Test of Playfulness (ToP), and Sensory Processing Measure (SPM). Results: The sensory processing of children with ASD were related to decreased functional skills performance of ADL (F = 94.4, *p* = 0.00) and playfulness (*p* = 0.00) than neurotypical children; in addition, the problems of sensory reactivity were associated with worse development in these occupational areas (*p* = 0.00 for both Spearman correlations). Conclusions: Children with ASD present worse performance of functional skills and playfulness than neurotypical ones. Likewise, sensory reactivity is related to the development in the occupational areas.

## 1. Introduction

Recent studios about the Autism Spectrum Disorder (ASD) prevalence, point out big differences depending on the area studied; however they agree on it has been detected a significant increase of diagnosed cases of ASD [1,2].

The problems of sensory processing in people with ASD are well known [3,4]. The Diagnostic and Statistical Manual of Mental Disorders of the American Psychiatric Association DSM-5 [5], states that this group presents sensory processing difficulties and sensory integration challenges, which usually affects more than one sensory modality and can include hypo (when the child needs a great intensity of a certain stimulus to be able to perceive, register and generate an adaptive response) or hyper-reactivity (when the child overreacts to a weak stimulus) to certain sensory stimulus; in addition, the study of Sanz-Cervera et al. [6] pointed out that those problems are present in different contexts. Along those lines, three types of insufficient sensory processing may be identified in the group of children with ASD: the first is due to incorrectly registered sensory input in the mind of the child [4,7]; another second supposition is because of poor modulation/reactivity of the sensory input [8,9]; and a third reason is due to disinterest in new, proactive and constructive occupations [7,8].

These alterations affect and interfere with the quality of life of individuals with ASD, for which reason it is fundamental that they are taken into account during the process of evaluation and diagnosis and when planning interventions and support, to promote participation and independence among people with ASD in social, educational, workplace, and community life [10].

The occupational performance of a person, and therefore his performance in Activities of Daily Living (ADL), is affected when that person presents an alteration of sensory processing [11]. Likewise, the difficulties over detecting, processing, interpreting, and finding an adaptive response to the sensory stimuli interfere with the participation of children in significant occupational activities, such as play.

The aims of this study are, on the one hand, to identify whether exist differences between children with ASD and neurotypical developmental in relation to independence in ADL and playfulness, as well as to confirm the differences with regard to sensory processing and integration challenges; and, on the other, to confirm whether a relation exists between independence in ADL, playfulness, and sensory processing.

## 2. Materials and Methods

### 2.1. Type of Study/Design

A cross-sectional descriptive study design was employed in this study.

### 2.2. Participants

The study sample was obtained by using non-probabilistic convenience sampling. Two types of samples were recruited, 20 children with a diagnosis of ASD, and 20 with neurotypical development. The inclusion criteria to form part of the group of children with ASD were: (a) to have a clinical diagnosis of ASD according to DSM-5 criteria; (b) to be aged between 4 and 10 years old; and, (c) to have had an informed consent form signed by a parent or legal guardian. On the other hand, the exclusion criteria was (a) the presence of comorbid disorders added to ASD. The inclusion criteria established for children with neurotypical development were: (a) to be aged between 4 and 10 years old, and (b) having had an informed consent form signed by a parent or legal guardian. In turn, the exclusion criterion was the presence of pathologies or disabilities. Starting from the base and taking as a reference the DSM-5 [5], where it is stated that the presence of hyper or hypo-sensory reactivity is characteristic of ASD.

### 2.3. Procedure

The Burgos University Ethic Committee approved the study with the code IR-6/2018 on 27 February 2018. The parents of the children in the sample were contacted, and having completed the informed consent form; three evaluation scales-Pediatric Evaluation of Disability Inventory (PEDI), Test of Playfulness (ToP), and Sensory Processing Measure (SPM) were administered to the father/mother or caregivers of the children during an interview: in a personal interview of an approximate duration of one hour and a quarter. Likewise, a series of sociodemographic data were also collected.

### 2.4. Assessment Tools

The following tools were administered as evaluation instruments.

#### 2.4.1. Pediatric Evaluation of Disability Inventory (PEDI)

Pediatric Evaluation of Disability Inventory (PEDI) [12]. This clinical evaluation instrument was developed by Haley, Andrellos, Coster, Haltiwanger, and Ludlow in 1992. Applied through a structured interview that targets the principal caregivers of the children, its duration is between 45 and 60 min. It is formed of two scales: one that evaluates Functional Skills (FS) and that has 197 items; and another formed of 20 items to evaluate caregiver help and modifications to the environment and equipment that the child needs [13]. The first scale, FS, is in turn divided into three domains: personal care, with 73 items; mobility, formed of 59 items; and social functioning, composed of 65 items. The score for each item of this scale corresponds to 1 capable and 0 incapable. The total value is obtained by summing the scores of the items that comprise each domain. With regard to the second scale, the subscale of caregiver help has as its objective to quantify the level of assistance that the child requires from the adult during the performance of the activities; scoring from 0 totally dependent to 5 independent [13,14]. Likewise, if the child needs some change of environment or the use of some equipment for the development of those tasks it is registered on the sub-scale of modifications, under the parameters of none, child rehabilitation and extensive [13,14]. This instrument offers acceptable psychometric properties, both in its original version [12] and in its translation and validation in Castilian Spanish [13].

#### 2.4.2. Test of Playfulness (ToP)

Test of Playfulness (ToP) [15]. This scale of evaluation was developed by Bundy in 1997, with the objective of evaluating play in children from two to ten years old. The ToP is applied within a space of time of 15 min and provides information on four aspects of play: intrinsic motivation, suspension from reality, locus of internal control and coherent framework of play [16,17]. It is composed of 24 items, which reflect the extension, intensity and skill with which the child engages in playful activities. It is scored on a scale of 4 points (from 0 to 3), in which the options for response are: 0 rarely or never, 1 sometimes, 2 most of the time and 3 almost always [16]. It is a reliable test, with respect to psychometric properties, with response validity and concurrent validity [18].

#### 2.4.3. Sensory Processing Measure (SPM)

Sensory Processing Measure (SPM) [19]. A standardized instrument of evaluation developed by Parham, Ecker, Kuhaneck, Henry and Glennon in 2007; which is based on the theory of sensory integration of Ayres [20]. It evaluates sensory and behavioral functioning, as well as social participation in children of five to twelve years old [20]. The SPM Home Form version was used for this study [19], which evaluated the sensory functioning of the child in the home. It comprises 75 items and is administered to the parents of the children, with an administration time of between 15-and-20 min [21]. Each item scored on a 4-point Likert-type scale in which the options for response are: never, occasionally, frequently and always. The SPM Home Form comprises eight dimensions: *Social participation*, *Planning and ideas*, *Vision*, *Hearing*, *Touch*, *Taste and Smell*, *Body awareness*, and *Balance and motion*. The sensory systems were grouped within another dimension known as Total Sensory Systems (TOT). On the other hand, the dimensions of *Social participation* and P*lanning and ideas* guide evaluation of the functions of higher sensory integration. It is worth noting that the items of the dimensions TOT and *Planning and ideas* score higher when the systems are more affected and planning and organize movement is worse [22]. However, the better the social participation the higher score the dimension S*ocial participation*. Finally, this instrument offers some acceptable psychometric properties in all of its dimensions: high internal consistency (Cronbach’s alpha varied between 0.75 and 0.95) and an approximate validity of 0.5 [22].

### 2.5. Data Analysis

The statistical software package IBM SPSS Statistics version 24 was used to analyze the data. The statistical analysis was done as a function of the adjustment to normality and the number of the sample (Shapiro-Wilk test was applied to test the sample normality). In particular, the tests used were one way ANOVA, Chi squared, and the Spearman Correlation. The *p*-value was established less than 0.05. Raw data were used to analyze the SPM variables.

## 3. Results

### 3.1. Sample Analyses

In order to check if there were any differences between both groups, it was calculated the one way ANOVA test [23].

Given that, it is a non-probabilistic sample, and in order to check if the sample was homogeneous, there were analyzed different variables. Related to the age of the children and parents, as it is showed in Table 1, there is no differences between both groups.

Neither were differences appreciated with regard to gender, environment in which the children live and level of studies of the parents, following the application of the Chi squared test, except for gender, because 80% of the children with ASD from the sample were boys and 20% were girls, unlike the neurotypical children where half were boys and the other half girls (Table 2).

### 3.2. Inferential Analysis

One-way ANOVA tests were performed to identify differences in relation to performance in ADL and playfulness between children with ASD and neurotypical children. The test results demonstrated that children with ASD presented lower scores than the neurotypical children (less independence in the ASD), as well as their greater need for help from caregivers, more modifications in the environment and the use of support products (Table 3). Significant results (*p* = 0.000) were obtained in all the dimensions of the ToP scale that reflected the differences that exist between both groups of children in extension, intensity, and playfulness (Table 3).

With the purpose of testing for differences in relation to sensory processing, the dimensions of the SPM scale were compared between both groups and significant results (*p* = 0.000) were obtained in all dimensions of the SPM scale, which suggest the existence of differences in sensory reactivity between children with ASD and neurotypical children (Table 4). In the case of *Social participation*, interpretation is reversed with respect to the other dimensions, as according to the SPM manual, the higher the score, the better the social participation, unlike the other dimensions of the scale, in which a higher score corresponds to further problems in sensory systems and worse planning.

### 3.3. Correlation between Performance of ADL and Sensory Reactivity

A Spearman correlation was performed between the variables of PEDI, ToP, and *Social participation* and *Planning and ideas* with the TOT dimension of the SPM scale (Table 5), in order to test the hypotheses that point to the existence of a relation between sensory reactivity and ASD and playfulness, as well as S*ocial participation* in children with ASD. In all three cases, it was confirmed that the accentuation of sensory problems led to worse performance in ADL, playfulness and the *Social participation* of children with ASD (negative correlation). Moreover, in relation to *Planning and ideas* and sensory processing, a positive correlation indicated that the accentuation of sensory reactivity led to more problems arising in planning and ideas, all of it taking as its base the criteria of scores on the SPM scale.

## 4. Discussion

The purpose of this study was to identify whether differences exist between children with ASD and neurotypicals in relation to ADL, playfulness, and sensory processing. In relation to this purpose, the results showed that it seems to be a relation between ASD and having greater difficulties in the performance of ADL, which was previously reported by Kao et al. [24]. On the other hand, regarding playfulness in ASD, it has been studied by several authors, suggesting being related to the environment [25] and the pretend play [26]. Likewise, the results suggested that children with ASD experience problems of sensory reactivity and those problems are likewise related to worse performance in the two above-mentioned occupational areas. The results therefore agree with the diagnostic criteria established by the DSM-5 [5] for ASD: marked deficits in verbal and non-verbal social communication skills, related with problems in play and in social participation; restrictive and repetitive behavioral patterns, interests and activities, among which problems of hypo- and hyperactivity to sensory stimulus and unusual interest in sensory aspects of the environment; and the systems of ASD are causes of a clinically significant deterioration in social, occupational, and other important areas of functioning, related with difficulties in the performance of ADL obtained as a result of this study.

In agreement with our results, with respect to the worse performance in ADL among children with ASD, we find the study by Schaaf et al. [27], in which a group of children with sensory reactivity disorders, although not specifically ASD, were compared with a group of neurotypical children. The authors found that the problems of sensory reactivity interfered negatively in the production of adaptive responses and, in consequence, affected the proper performance of ADL, in communication and social skills. Likewise, in the study carried out by Case-Smith, Weaver and Fristad [28], after evaluating children with ASD and sensory difficulties, they concluded that the group that received occupational therapy treatment obtained better results for self-care, social skills, and functional independence.

In relation with the difficulties found for playfulness and the social participation of children with ASD, there is a study conducted by Evans [29], in which the early presence of sensory reactivity and awareness of novelty was examined with regard to the behavior of children in their peer groups. It was concluded that the sensorial reactive children appeared less sociable, more susceptible to isolation at play and were more susceptible to the use of instrumental aggression in their interactions with their colleagues. Likewise, the associations between sensory perception and the behavior of the children was observed to differ from other dimensions of temperament, which suggests that sensory reactivity contributes in a unique way to the understanding of behavior of children and their social and playful actions. In agreement, Roberts, Stagnitti, Brown and Bhopti [30] investigated the relation between sensory processing and playfulness in children and concluded that play involved the child through multiple sensory stimuli, which contributed in a positive way to development and sensory processing. They likewise upheld the efficacy of the use of play in occupational therapy as a means of working body awareness, balance, and touch, and to be able to promote social participation.

In parallel to the findings on the presence of sensory problems among children with ASD and their influence on occupational areas, the study conducted by Chang et al. [31] examined whether children with ASD and neurotypical children differed in their responses to various sensory stimuli and how these influenced their behavior. It was found that the children with ASD presented higher reactivity to auditory sensory stimuli, which in consequence negatively affected their behavioral conduct, interfering in their daily activities. Additionally, Schaaf and Case-Smith [32] carried out an investigation into sensory interventions in children with ASD and sensory problems and came to the conclusion that the therapy of sensory integration, provided with occupational therapists, was effective at improving functional and social skills and self-care.

As future lines of research, it is proposed to examine specific sensory systems (vision, hearing, touch…) in greater depth, to establish which involve greater affectation, and the extent to which these those systems affect each area of specific performance (food textures, brushing teeth, dressing…).

Although the study has limitations (small and non probabilistic sample, scarce information about cognitive and adaptative functioning, among others), the practical implications of the results could be oriented towards preparing occupational therapy guides on the sensory problems of ASD, as well as towards the proposal of solutions and interventions from the perspective of occupational therapy, under the sensory integration method, directed towards treating the sensory problems of this group, in order to favor performance in ADL and promote exploration and participation in play.

## 5. Conclusions

As conclusions to this study, it has been pointed out that children with ASD have more difficulties in performing ADL and playfulness than neurotypical children, and that this group need greater support from their caregivers, as much as they need modifications to the environment and the use of support products. Likewise, it has been suggested that sensory reactivity is involved and has relation with the performance of both occupational areas.

It is point out the importance of continuing to carry out scientific studies that evaluate the impact of sensory reactivity in different occupational areas and the efficacy of occupational therapy.

## Figures and Tables

**Table 1:** Data relating to the analysis of the quantitative variables of both groups.

Variables	ASD		Neurotypical		ANOVA		
	Mean	SD	Mean	SD	F	*p* Values	Ŋ^2^ Partial
Age of children (months)	88.15	24.384	84.65	21.695	0.230	0.634	0.006
Age of parents (years)	42.50	5.671	41.10	3.463	0.888	0.352	0.023

Note. SD: Standard Deviation; F: F-Statistic; Ŋ^2^ Partial: Partial Eta Squared; *p* values: Conditional probability that a relation exists; *: *p* < 0.05.

**Table 2:** Data relating to the analysis of the qualitative variables of both groups.

Variables		ASD		Neurotypical		Chi Squared		
		Number	%	Number	%	X^2^	gl	*p* Values
Gender	Male	16	40	10	25	3.956	1	0.047 *
	Female	4	10	10	25			
Environment	Urban	19	47.5	19	47.5	0.000	1	1.000
	Rural	1	2.5	1	2.5			
Level of studies of parents	Basic	2	5	5	12.5	7.734	4	0.102
	Baccalaureate	2	5	0	0			
	Further Educ.	3	7.5	2	5			
	Higher Educ.	11	27.5	6	15			
	Doctoral	2	5	7	17.5			

Note. %: of the total; X^2^: Chi Squared; gl: Degrees of Freedom. *p* values: Conditional probability that a relation exists; *: *p* < 0.05.

**Table 3:** Results of the differences between children with TEA and neurotypical children on the PEDI and the ToP scale.

Variables		ASD		Neurotypical		ANOVA		
		Mean	SD	Mean	SD	F	*p* Values	Ŋ2 Partial
PEDI	FS	121.25	28.38	185.65	8.554	94.403	0.000 *	0.713
	FS. CP	42.50	14.489	68.20	3.679	59.108	0.000 *	0.609
	FS. Mobility	50.40	5.133	57.45	1.877	33.266	0.000 *	0.467
	FS	28.35	11.249	60.00	3.741	142.538	0.000 *	0.790
	Help from caregiver	61.35	15.363	95.25	3.537	92.476	0.000 *	0.709
	Modifications	52.75	2.971	59.25	0.85	88.457	0.000 *	0.700
ToP	Extension	19.90	7.475	44.15	4.145	160.956	0.000 *	0.809
	Intensity	5.70	2.735	10.25	1.712	39.742	0.000 *	0.511
	Skill	5.50	4.00	21.80	2.667	229.355	0.000 *	0.858

Note. SD: Standard Deviation; F: F-Statistic; Ŋ2 Partial: Eta Partial Squared; *p* values: Conditional probability that a relation exists; *: *p* < 0.05.

**Table 4:** Results of the differences between children with ASD and neurotypical children on the SPM scale.

Variables SPM	ASD		Neurotypical		ANOVA		
	Mean	SD	Mean	SD	F	*p* Values	Ŋ2 Partial
TOT	112.70	22.98	63.15	9.80	78.661	0.000 *	0.674
Vision	24.45	5.853	13.75	3.354	50.314	0.000 *	0.570
Hearing	19.35	5.214	10.70	2.079	47.487	0.000 *	0.555
Touch	22.70	5.849	12.75	2.221	50.569	0.000 *	0.571
Body awareness	20.80	6.321	12.40	2.479	30.608	0.000 *	0.446
Balance and movement	20.75	6.298	11.35	2.134	31.909	0.000 *	0.456
Social participation	21.40	3.871	36.55	3.017	190.538	0.000 *	0.834
Planning and ideas	26.85	5.363	10.40	1.465	175.073	0.000 *	0.822

Note. SD: Standard Deviation; F: Value of the F Statistic; Ŋ2 Partial: Partial Squared Eta; *p* values: Conditional probability that a relation exists; *: *p* < 0.05.

**Table 5:** Results of the relation between performance in DLA and sensory reactivity.

Variables		TOT	
		Spearman Correlation	*p* Values
PEDI	FS	−0.858	0.000 *
	FS. BA.	−0.855	0.000 *
	FS. Mobility	−0.754	0.000 *
	FS	−0.850	0.000 *
	Caregiver help	−0.859	0.000 *
	Modifications	−0.840	0.000 *
ToP	ToP Extension	−0.803	0.000 *
	ToP Intensity	−0.743	0.000 *
	ToP Skills	−0.797	0.000 *
SPM	Social Participation	−0.855	0.000 *
SPM	Planning and ideas	0.829	0.000 *

*p* values: Conditional probability that a relation exists; *: *p* < 0.05.
